# Influence of BOF and GGBFS Based Alkali Activated Materials on the Properties of Porous Concrete

**DOI:** 10.3390/ma12142214

**Published:** 2019-07-10

**Authors:** Wen-Ten Kuo, Yi-Syuan Gao, Chuen-Ul Juang

**Affiliations:** Department of Civil Engineering, National Kaohsiung University of Science and Technology, Kaohsiung 807, Taiwan

**Keywords:** basic oxygen furnace slag, porous concrete, ground granulated blast furnace slag based alkali activated materials binder, liquid-to-solid ratio (L/S), pore filling paste ratio

## Abstract

In this study, environmentally friendly ground granulated blast furnace slag (GGBFS) based alkali activated materials and basic oxygen furnace slags (BOFs) were used as bonding materials and aggregates, respectively, to produce novel, environmentally friendly GGBFS based porous concrete. Porous concrete with a particle size of 4.75–9.5 mm and 9.5–19.00 mm was used as an aggregate. The “liquid-to-solid ratios” (L/S) variable was set at set at 0.5 and 0.6, and the “percentage of pore filling paste ratio” variable was controlled at 40%, 50%, and 60%. The curing period was set at 28 d, and the relationship between connected porosity and permeability, as well as that between unit weight and the pore filling paste ratio percentage were explored using analysis of variance. The results showed that the porous concrete had a maximum compressive strength of 8.31 MPa. The following results were obtained. An increase in percentage of pore filling paste ratio increased compressive strength. Permeability was measured at 4.67 cm/s and was positively correlated with porosity. An increase in porosity increased permeability, in which porosity was positively correlated with the percentage of pore filling paste ratio. The maximum splitting strength achieved during the 28 d was 1.46 MPa, showing a trend similar to that of compressive strength.

## 1. Introduction

Porous concrete—made from zero or traces of fine aggregates, as well as a mixture of coarse aggregates, water, and insufficient cement—is an environmentally friendly paving material [1] that features favorable permeability. In addition, it can be used to regulate the surface temperature of roads and possesses a connected porosity of approximately 20% [2]. According to the US Green Building Council to promote green building assessment, porous concrete can reduce the heat island effect on the environment [3] and can be filtered according to water quality for potential re-use [4]. In addition, porous concrete features a porosity and permeability higher than those of regular concrete, enabling rain to be quickly discharged [5]. The compressive strength of porous concrete is primarily decided by the concrete’s porosity, which is determined by its aggregate size, aggregate shape, and water-cement ratio. Crouch et al. [6] found that using well-graded aggregates created higher compressive strength and porosity. At a low water-cement ratio and an aggregate particle size of 12.5 mm or 5 mm, the cement slurry can effectively eliminate gaps between aggregate fillings [7]. Porous concrete features a porosity between 18%–35% and a compressive strength between 2.8–28 MPa [8]. Park and Tia [9] studied the water purification ability of porous concrete, in which they found that porous concrete with a relatively smaller aggregate particle size and a relatively higher porosity demonstrated the filtering capability of removing the total phosphorus and total nitrogen in water. The reason was that aggregates have a relatively larger specific surface area.

Basic oxygen furnace slag (BOF) is a byproduct created during the steelmaking process and is the solid waste created when iron ore are converted in a basic oxygen furnace [10]. BOF features polygonal-shaped aggregates, high specific gravity, high unit weight, low water absorption rate, low abrasion rate, and high compression resistance [11]. Each year, Taiwan produces approximately 1.6 million tons of BOF, accounting for 25% of its general solid waste. Therefore, properly using BOF can elevate the reuse of industrial waste, reduce pollution and damages to the environment, help protect natural resources [12], and increase profits. BOF treatment and processing-related technologies are now mature. Over the past few decades, they have replaced natural aggregates in road construction [13,14] and are used as the mixture of coarse aggregates and various types of asphalt in roads [15]. Mohammad et al. [16] studied to develop an alkali-activated concrete containing carbonated basic oxygen furnace (BOF) slag aggregates. The results showed that using carbonated BOF slag aggregates led to higher strength than using aggregates in alkali activated concretes. Palankar et al. [17,18] investigated the partial and full replacement of natural aggregate with carbonated BOF aggregates (by weathering for 6–9 months) in alkali-activated granulated blast furnace slag (GGBFS)/fly ash concrete. Additionally, the utilisation of carbonated BOF aggregates (through a carbonation reactor) as a partial replacement for natural aggregates in cementitious mortars was studied in [19].

Alkali activated materials, introduced by French scientist Joseph Davidovits in 1978, are a new, green, and environmentally friendly material that has emerged in recent years. These materials are primarily made by placing aluminum silicate materials in an overbased alkaline solution to produce silicon and aluminum colloids, which are then dehydrated and hardened to form interconnected [SiO_4_]^4−^ and [AlO_4_]^5−^ tetrahedrons. Alkali activated materials display excellent fire-resistance, heat-insulation, and acid and base-resistance abilities, as well as mechanical properties [20,21,22,23,24,25,26]. In addition, they are a zeolite-like aluminum silicate material with a three-dimensional structure, a type of early-high material, and capable of achieving high compressive strength within a short period. Alkali activated materials and fly ash added elements, such as chloride ions and calcium sulfate, can show high compressive strength.

Yeih et al. [27] manufactured general porous concrete by using oxidized slags and natural aggregates, in which the porous concrete showed a permeability coefficient and a compressive strength higher than those of the gravel-made porous concrete. An increased particle size also caused the British pendulum number (BPN) to drop; this signified a drop in friction and may have been caused by the electric arc furnace slag having a coarser surface than that of gravel, which increased friction. Kuo and Shu [15] made porous concrete by washing the incineration subballast and natural aggregates. Results showed that this porous concrete had a compressive strength of 4.75–12.68 MPa, a flexural strength of 1.76–3.11 MPa, a splitting strength of 0.51–1.40 MPa, and a maximum splitting and flexural strength that were 1/9 and 1/4 of its compressive strength, respectively. An increase in the percentage of pore filling paste ratio decreased concrete porosity and permeability but increased concrete strength. Park and Tia [9] created porous concrete by adding silicate cement to natural aggregates and found that the use of aggregates with a smaller particle size and porous concrete with a higher porosity effectively removed the phosphorus and nitrogen content in water. They attributed this result to the porous concrete having a higher specific surface area in contact with water. When using gangue and aluminum hydroxide to make a permeable brick, the permeability coefficients ranged from 1.37 cm/s to 1.55 cm/s. Jang et al. [28] developed environmentally friendly porous concrete by using fly ash and high furnace slag-based alkali activated materials and found that the said porous concrete had a total porosity greater than that of the general porous concrete and that the heavy metal dissolution of bottom ash alkali activated materials exhibited a definite effect on porosity. Chang et al. [29] built porous concrete by using electric arc furnace slags and alkali activated materials and found that at a pore filling paste ratio of 90% and a particle size of 0.24–0.48 cm, the porous concrete displayed a compressive strength and permeability of 35 MPa and 0.49 cm/s, respectively, in 28 d. A BPN of 79 indicates favorable slip resistance. Chang also found that the porous concrete displayed a mechanical strength stronger than that of regular Portland cement. Tho-in et al. [30] used a natural aggregate and a fly ash-based inorganic polymer made of permeable concrete. The results showed a porosity between 28.7%–34.4%, a permeability coefficient between 1.92–5.96 cm/s, and a compressive strength between 5.4–11.4 MPa, with better water permeability and good compressive strength, similar to the Puntan permeable concrete. Zaetang et al. [31], using bottom ash and inorganic polymer made of permeable concrete, showed that when the sodium hydroxide concentration increased from 10 M to 15 M, its compressive strength increased to 5.7–8.6 MPa. In addition, with a Curing temperature of 60 °C to 120 °C, the compressive strength also increased. At 90 °C, the strength increased significantly.

## 2. Testing Methods

### 2.1. Materials and Mix Proportions

This study used BOF as aggregates for making environmentally friendly porous concrete. The particle sizes used were 4.75–9.5 mm and 9.5–19.0 mm, and the physical and chemical properties of the BOF are shown in Table 1 and Table 2. The bonding materials consisted of ground granulated blast furnace slag (GGBFS), water glass, and sodium hydroxide, in which deionized water was added to the sodium hydroxide to produce an aqueous solution with a concentration of 5 N.

Because no standardized ratio and design method are currently available for making porous concrete, the volumetric method was adopted. This method involves first finding the porosity, relative density, and unit weight of BOF with particle sizes of 4.75–9.5 mm and 9.5–19.0 mm. The porosities of the two different aggregates are shown in Table 1, in which the numbers denote the gap between the aggregates. The gaps are filled using alkali activated materials of different percentages. The design proportion of the porous concrete is shown in Table 3.

### 2.2. Methods

In this study, a cylindrical specimen that measured 10 cm and 20 cm in diameter and height, respectively, was used. Subsequently, a bonding material (i.e., GGBFS) and the BOF were placed inside a mixer and mixed for 1 min. Next, an alkali solution was slowly added, and the mixture was mixed for 2 min. Then, porous concrete was introduced and rammed. After the porous concrete was thoroughly mixed, a unit weight experiment was performed using the American Society for Testing and Materials (ASTM) C138 [32], and a hardness test was administered after a curing period of 28 d (the compressive, splitting, and flexural strengths were measured using the ASTM C39 [33], ASTM C496/C496M [34], and ASTM D790 [35], respectively). A permeability test was conducted using the fixed head method, whereas slip resistance was measured using the British pendulum tester (skid resistance and friction tester A113-01, MATEST, Arcore (MB), Italy) [36].

## 3. Results and Discussion

### 3.1. Connected Porosity

**Figure 1:**
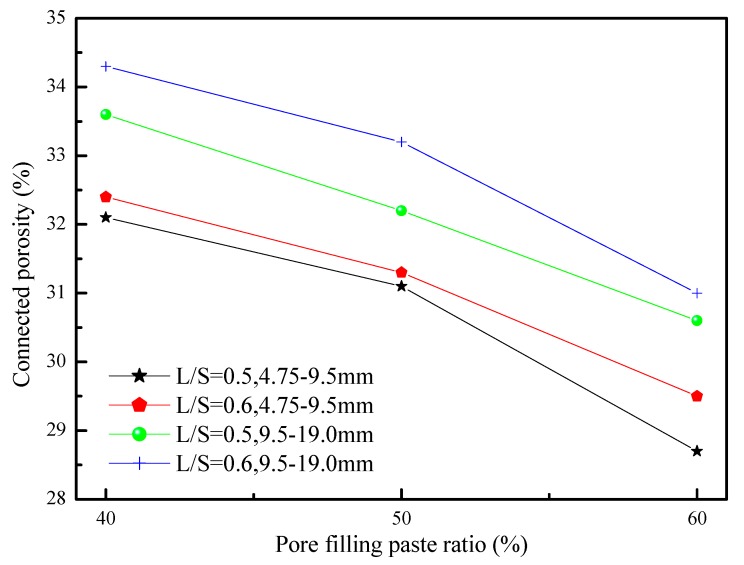
Effect of the pore filling paste ratio on the connected porosity for porous concrete.

The connected porosity and permeability of porous concrete are correlated. Figure 1 shows the relationship between the connected porosity and percentage of pore filling paste ratio of novel, environmentally friendly GGBFS-based porous concrete. By investigating the porous concrete of two different particle sizes, the present study found that the connected porosity of porous concrete with a particle size of 9.5–19.0 mm was higher than that with a particle size of 4.75–9.5 mm. This was because the gap between the aggregates in the first porous concrete was larger than that between the aggregates in the second porous concrete. Therefore, when the percentage of pore filling paste ratio was kept constant, the porous concrete with a particle size of 9.5–19.0 mm demonstrated a connected porosity higher than that with a particle size of 4.75–9.5 mm. An increase in the percentage of pore filling paste ratio decreased the connected porosity of the porous concrete, which was because the rise in the percentage of pore filling paste ratio resulted in the pores behind being filled by alkali activated materials.

**Figure 2:**
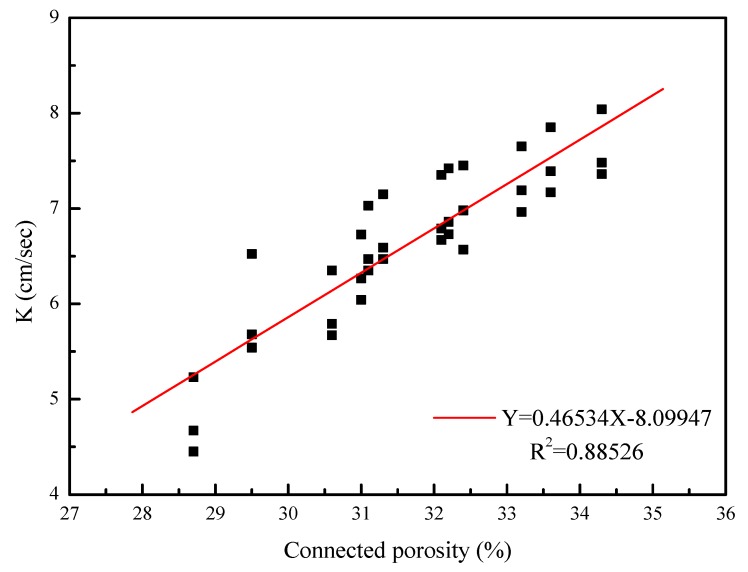
Effect of connected porosity on the permeability coefficient for porous concrete.

Figure 2 shows the relationship between the connected porosity and permeability, in which permeability was positively correlated with connected porosity for both aforementioned types of porous concrete. Under identical connected porosity conditions, porous concrete with a smaller aggregate particle size had a smaller connected pore sectional area and a more curved permeable path inside the specimen. Conversely, porous concrete with a larger aggregate particle size had a larger connected pore sectional area and a straighter permeable path inside the specimen. The permeability coefficient increases with an increase in porosity. When the permeability increased to 7.48 cm/s, the porosity was more than 34%, and when the porosity was about 4.67 cm/s, the porosity increased to 28%. The equation is equal to Y = 0.46534X − 8.09947, while the porosity and permeability coefficient is proportional to the values shown in Figure 2. Porosity is an important factor affecting the permeability coefficient.

### 3.2. Unit Weight

**Figure 3:**
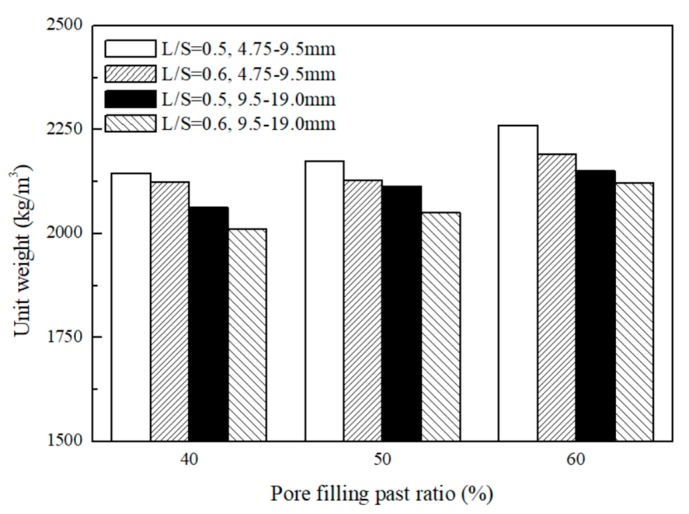
The unit weights for porous concretes.

The novel environmentally friendly GGBFS-based porous concrete had a unit weight of approximately 2011–2260 kg/m^3^ (Figure 3), which shows the relationship between unit weight and pore filling paste ratio percentage. For porous concrete of a single particle size, the unit weight was positively correlated with percentage of pore filling paste ratio and negatively correlated with liquid-to-solid ratio (L/S). In addition, when the liquid-to-solid ratio was held constant, an increase in aggregate particle size decreased the porous concrete’s specific surface area, which decreased the unit weight. This was because the BOF had a specific gravity of approximately 3.30 greater than the approximately 2.65 specific gravity of the natural aggregate. Porous concrete made from the GGBFS-based alkali activated slag cement had a unit weight greater than that of general porous concrete (1400 kg/m^3^–1900 kg/m^3^).

### 3.3. Permeability

**Figure 4:**
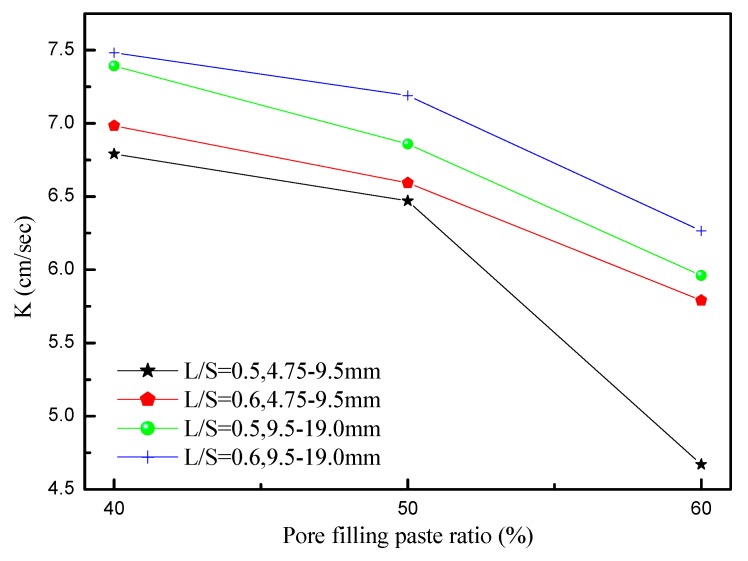
The influences of the pore filling paste ratio on the water permeability of porous concrete.

The permeability coefficient is a crucial indicator of the permeability of concrete. According to the Japan Concrete Road Association, Tokyo, Japan (1996), porous concrete must feature a permeability greater than 0.01 cm/s. Figure 4 shows the relationship between the permeability and pore filling paste ratio percentage over 28 d, which showed that porous concrete with a particle size of 9.5–19.0 mm displayed a higher permeability than that with a particle size of 4.75–9.5 mm. This was because the former created larger pores than the latter during the stacking process. The permeability of the two types of porous concrete ranged between 4.67 and 7.48 cm/s during the 28 d. At a liquid-to-solid ratio of 0.5 and a pore filling paste ratio percentage of 60%, porous concrete with a particle size of 4.75–9.5 mm produced a permeability of 4.67cm/s. This low permeability was a result of the particle size being smaller and more pores between the aggregates being filled. At a liquid-to-solid ratio of 0.6 and a percentage of pore filling paste ratio of 40%, porous concrete with a particle size of 9.5–19.0 mm showed a maximum permeability of 7.48 cm/s. In addition, when the pore filling paste ratio percentage was held constant, pores created by porous concrete (with a particle size of 4.75–9.5 mm) during the stacking process were regularly and completely filled by the slurry, causing the permeable path to fill up and the permeability to drop. An increase in aggregate particle size increased permeability. When the pore filling paste ratio increased, the number of pores that remained unfilled decreased, which made the pores highly sensitive to the slurry and prone to being completely filled by the slurry. This process then caused the permeability to decline.

**Figure 5:**
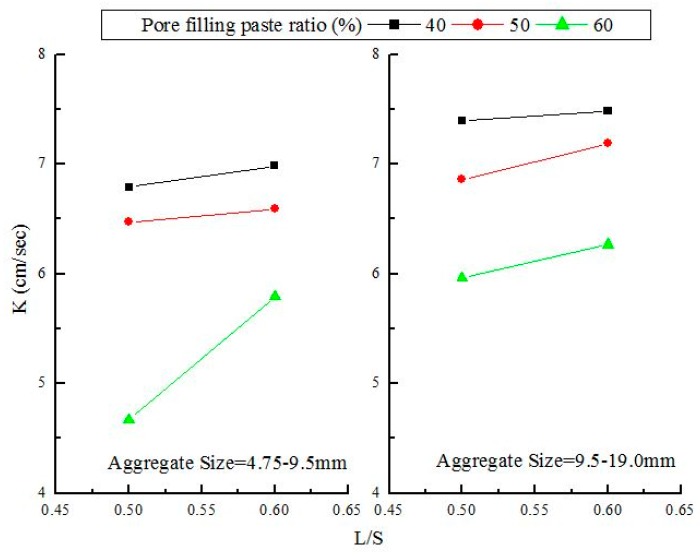
Effect of liquid-to-solid ratio (L/S) and aggregate size on the water permeability of porous concrete.

Figure 5 shows the effects of the liquid-to-solid ratio and particle size on permeability. The permeability of porous concrete with a particle size of 9.5–19.0 mm was greater than that of porous concrete with a particle size of 4.75–19.0 mm. In particular, at a liquid-to-solid ratio of 0.6 and a pore filling paste ratio percentage of 40%, the former displayed a permeability of 7.48 cm/s, which was the largest among all particle combinations. By contrast, at a liquid-to-solid ratio of 0.5 and a pore filling paste ratio percentage of 60%, the latter produced a permeability of 4.67 cm/s, which was the smallest among all particle combinations. For both types of porous concrete, permeability decreased as the amount of slurry increased. This result shows that the increase in slurry filled up a higher ratio of permeable paths in the specimen, causing the permeability to drop. In addition, the permeability of the porous concrete increased as the liquid-to-solid ratio increased, because, at low liquid-to-solid ratio, the slurry had higher viscosity and completely wrapped the aggregate particles. As the specimen hardened, the slurry-wrapped aggregates formed complete particles that remained in point-to-point contact with each other. This structure made the pores into permeable paths. Conversely, at high liquid-to-solid ratio, the slurry wrapped the aggregate particles but most failed to remain in point-to-point contact with each other because of high fluidity. As a result, permeable paths were less likely to form and permeability was lowered.

### 3.4. Compressive Strength

**Figure 6:**
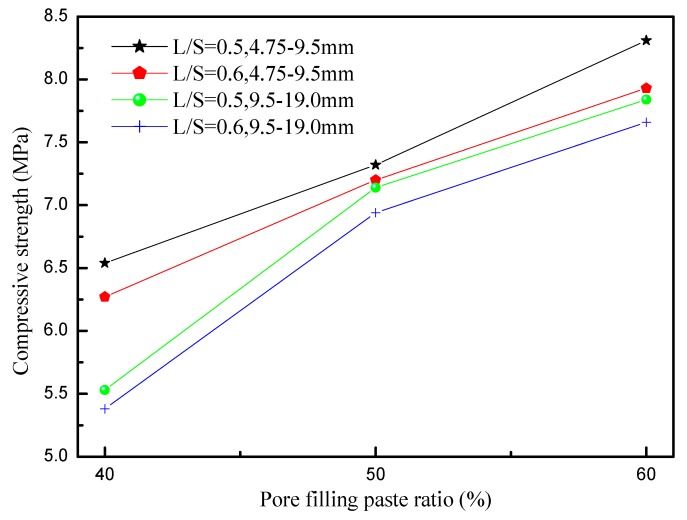
Compressive strengths for porous concretes using different aggregate size and L/S.

The compressive strength of the porous concrete measured in the 28 d was the most critical indicator of its mechanical properties. In general, typical porous concrete has a compressive strength of 2.8–28 MPa. Because porous concrete has many internal pores, its compressive strength is lower than that of general concrete. In this study, the compressive strength of the porous concrete in the 28 d was 5.38–8.31 MPa, which satisfied general porous concrete intensity requirements. Figure 6 shows the effect of the pore filling paste ratio percentage on compressive strength. Porous concrete with a particle size of 4.75–9.5 mm exhibited a stronger compressive strength than that with a particle size of 9.5–19.0 mm, whereas the latter displayed a bigger porosity than that of the former, showing that an increase in porosity decreased compressive strength. At identical aggregate particle sizes and liquid-to-solid ratio, compressive strength increased as the pore filling paste ratio percentage increased. Pore size was determined by the pore filling paste ratio percentage: when the pore filling paste ratio percentage increased, more aggregate pores were filled, resulting in a stronger intensity.

**Figure 7:**
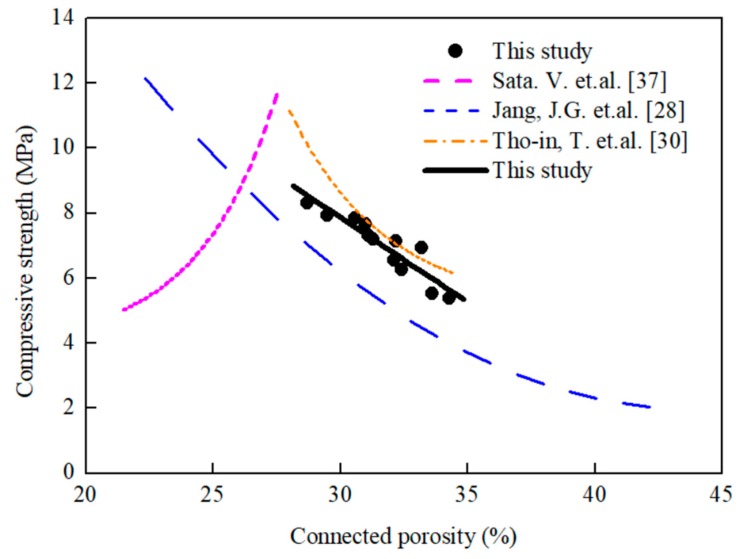
Measured connected porosity versus compressive strength.

Figure 7 shows the relationship between compressive strength and connected porosity. In the study of Jang et al. [28], connected porosity was 24.1%–0.5%, and maximum compressive strength was 9.5 MPa. In the study, compressive strength was 5.4–11.4 MPa, the maximum compressive strength was 11.4 MPa, and the connected porosity was 28.7 [30]. In the Sata et al. [37] study, when the porosity was 21.7%, the compressive strength was 4 MPa, the maximum pore size was 26.9%, and the compressive strength was 10 MPa. In this study, the GGBFS-based porous concrete had a connected porosity of 28.4%–34.3% and a compressive strength of 5.38–8.31 MPa. These results are similar to those presented in [28]. That is, the porosities were relatively higher, the compressive strengths were all lower than 10 MPa, and when the connected porosity increased, compressive strength decreased.

### 3.5. Splitting Strength

**Figure 8:**
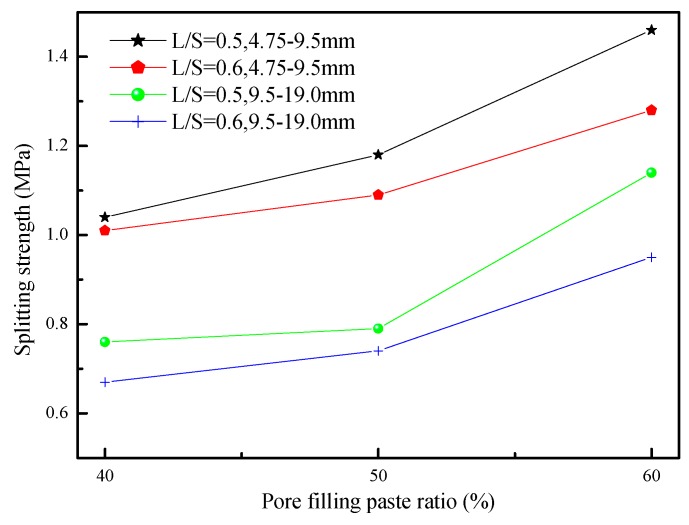
The splitting strengths for porous concretes using different aggregates size and L/S.

Figure 8 shows the relationship between the splitting strength and pore filling paste ratio of porous concretes. When the pore filling paste ratio was 40%, the splitting strength was 0.76 MPa; the pore filling paste ratio was 60%, and maximum splitting strength was 1.46 MPa. This result shows that the pore filling paste ratio was positively correlated with splitting strength. In addition, the maximum and minimum splitting strengths also occur in the minimum and maximum particle size, which shows that the development of splitting strength is inversely proportional to the size of the particle. When the pore filling paste ratio is 60%, and particle size is 4.75–9.5 mm, then the splitting strength is the largest. The highest splitting strength is 1/9 of the compressive strength.

**Figure 9:**
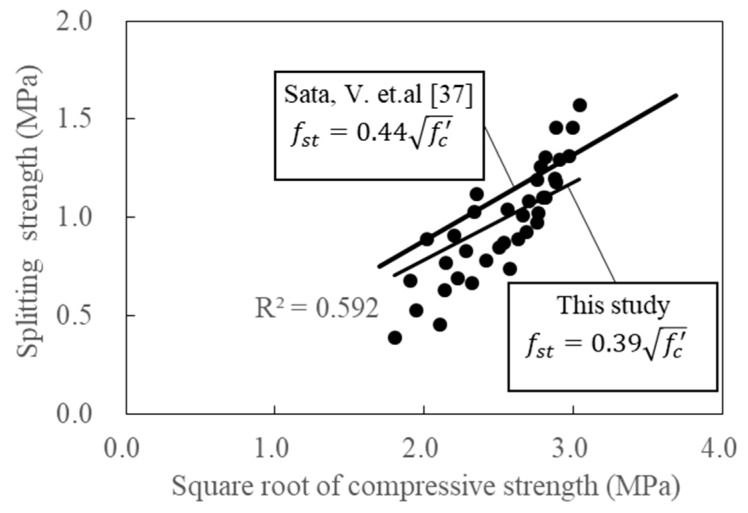
Relationship between the compressive strength and splitting strength of porous concretes.

Figure 9 shows the relationship between splitting strength and the square root of compressive strength. The results were subsequently compared with those obtained by Sata et al. [37], from which Equation (1) was derived. This equation shows that an increase in compressive strength increased splitting strength. However, the splitting strengths of the GGBFS-based porous concrete (attained via linear regression calculations) obtained in this study were lower than those found in the study of Sata et al. [37].

(1) $$f_{st}=0.39\sqrt{f_{c}^{\prime }}$$

### 3.6. Flexural Strength

**Figure 10:**
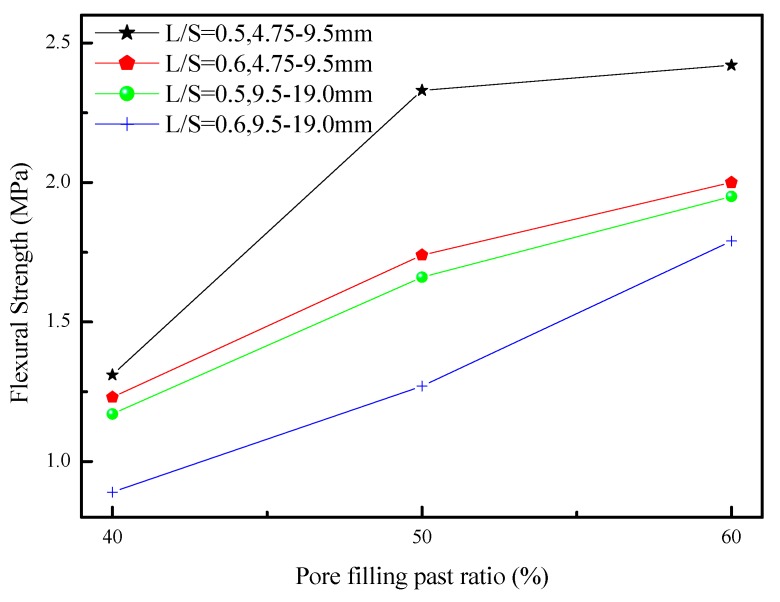
The flexural strength for porous concretes using different aggregates size and L/S.

Figure 10 shows the relationship between the flexural strength and pore filling paste ratio of porous concretes. When the pore filling paste ratio increased from 40% to 60%, the flexural strength of the GGBFS-based porous concrete increased, whereas an increase in the liquid-to-solid ratio decreased the flexural strength of the GGBFS-based porous concrete. This was because an increase in the liquid-to-solid ratio diluted the alkali activator solution, which lowered solution activity. By contrast, an increase in pore filling paste ratio increased the overall flexural strength of the GGBFS-based porous concrete and solved the problems that existed between the aggregates and slurry porosities. The trend of flexural strength was similar to that of the compressive strength.

**Figure 11:**
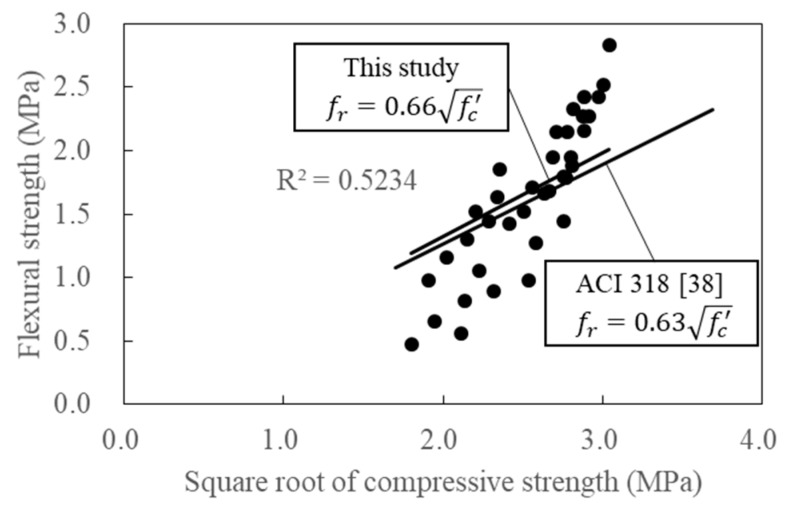
Relationship between compressive strength and flexural strength of porous concretes.

The linear regression of the compressive strength at the flexural strength and the radish ratio of 0.5 and 0.6 is shown at 7 days, 28 days, and 56 days of age in Figure 11. The compressive and splitting strengths of the furnace slag-based porous concrete were compared with the ACI 318 [38], from which Equation (2) was derived, showing that compressive strength was positively correlated with flexural strength.

(2) $$f_{r}=0.66\sqrt{f_{c}^{\prime }}$$

### 3.7. British Pendulum Test

**Figure 12:**
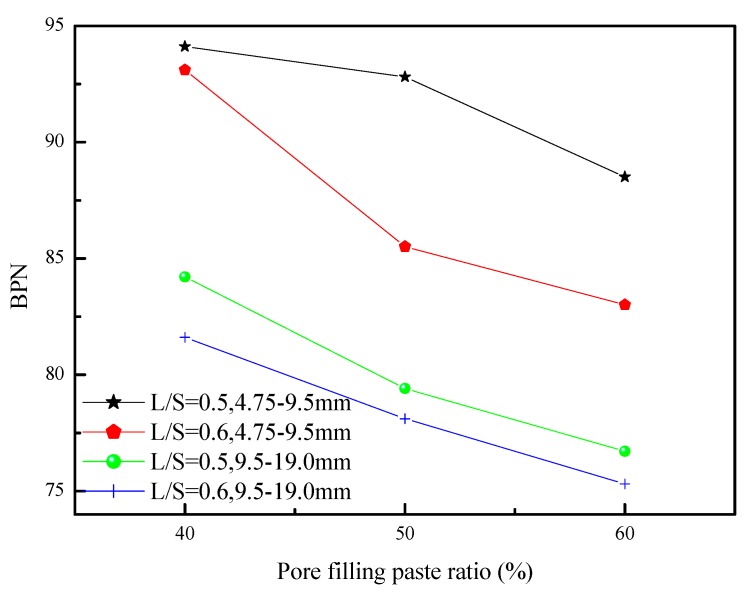
The British pendulum number (BPN) for porous concretes using different aggregates sizes and L/S.

The British pendulum tester can be used as an indicator of the slip resistance of porous concrete. A low BPN indicates poor slip resistance. Figure 12 shows the relationship between BPN and the pore filling paste ratio. Table 4 shows the recommended minimum BPN values obtained from a study report of Wessex Engineering Ltd. In the present study, the BPN values were 75.31–94.11 for all component ratios used, satisfying the aforementioned BPN value requirement. When the pore filling paste ratio increased, the BPN decreased. This may have been caused by the forming of a smoother surface as the slurry increased. The optimal BPN value was achieved when the pore filling paste ratio and particle size were 40% and 4.75–9.5 mm, respectively. Moreover, the porous concrete made using a particle size of 4.75–9.5 mm was larger than that made using a particle size of 9.5–19.0 mm. This may have been caused by the former having a rougher surface that subsequently increased the level of friction. This result shows that an increase in aggregate particle size decreased BPN.

The correlations between pore filling paste ratio, liquid-to-solid ratio, and compressive strength were estimated using an analysis of variance. A table of the mathematical models used in the regression analysis is shown in Table 5, indicating that when the α and BOF particle size were 0.05 and 4.75–9.5 mm, respectively, F = 12.03 < F_1,1,0.05_ = 18.51 and F = 173.86 > F_1,2,0.05_ = 19. Conversely, when the BOF particle size was 9.5–19.0 mm, F=16.47 < F_1,1,0.05_ = 18.51 and F = 264.51 > F_1,2,0.05_ = 19. This result indicates that the pore filling paste ratio had a stronger effect on GGBFS-based porous concrete.

## 4. Conclusions

1. The novel environmentally friendly GGBFS-based porous concrete had a connected porosity of 28.7%–34.3%, in which the connected porosity of GGBFS-based porous concrete with a particle size of 9.5–19.0 mm exceeded that of GGBFS-based porous concrete with a particle size of 4.75–9.5 mm. Compressive strength decreased and increased as the particle size and percentage of pore filling paste ratio increased and increased, respectively.
2. The optimal component ratio was achieved when the pore filling paste ratio and aggregate particle size were 60% and 4.75–9.5 mm, respectively. It yielded a compressive strength and permeability of 8.31 MPa and 4.67 cm/s, respectively, in 28 days.
3. The permeability coefficient and connected porosity of the novel, environmentally friendly GGBFS-based porous concrete decreased as the pore filling paste ratio increased. The permeability coefficient and connected porosity can be represented by the equation Y = 0.46534X – 8.09947.
4. The compressive and splitting strengths of the novel environmentally friendly GGBFS-based porous concrete decreased as permeability increased. The use of porous concrete with a particle size of 4.75–9.5 mm increased mechanical strength but decreased permeability because the percentage of filling increased.

## Figures and Tables

**Table 1:** Basic properties of basic oxygen furnace slag (BOF) used in the experiment.

Properties	Grade (mm)	
	4.75–9.5	9.5–19.0
Specific gravity (oven dry)	3.31	3.36
Absorption (%)	1.83	1.18
Unit weight (kg/m^3^)	1964	1991
Void content (%)	39.7	40.5

**Table 2:** Chemical composition of the BOF.

Chemical Characteristics (%)								
Item	SiO_2_	Al_2_O_3_	FeO	CaO	MgO	P_2_O_5_	MnO	CaO/SiO_2_
BOF	20.34	1.4	20.92	36.38	5.95	3.26	2.57	1.79

**Table 3:** Mix proportion of porous concrete.

Grade (mm)	L/S	Pore Filling Paste Ratio (%)	Mix Proportion(kg/m^3^)			
			BOF	GGBFS	NaOH	Na_2_SiO_2_
4.75–9.5	0.50	40	1964	134	20	47
		50	1964	167	25	58
		60	1964	201	30	70
	0.60	40	1964	117	21	49
		50	1964	147	26	62
		60	1964	176	32	74
9.5–19.0	0.50	40	1991	136	20	48
		50	1991	170	26	60
		60	1991	204	31	72
	0.60	40	1991	120	22	50
		50	1991	150	27	63
		60	1991	180	32	75

**Table 4:** Suggested minimum BPN for various conditions [27].

Conditions	BPN
Curve road, roundabouts, inclined slope	65
Common highway with traffic flow greater than 2000 vehicles/day	55
Others	45

**Table 5:** Analysis of variance (ANOVA) parameters for compressive strength of porous concrete.

Grade (mm)	Error Sources	Sum of Square	Degree of Freedom	Sum of Mean Squares	F	Threshold
4.75–9.5	L/S	0.10	1	0.10	12.03	18.51
	Pore filling paste ratio	2.93	2	1.47	173.86	19
	Random	0.02	2	0.01	-	-
	Sum	3.05	5	-	-	-
9.5–19.0	L/S	0.14	1	0.14	16.47	18.51
	Pore filling paste ratio	4.61	2	2.31	264.51	19
	Random	0.02	2	0.01	-	-
	Sum	4.77	5	-	-	-
